# A transcriptional hub integrating gibberellin–brassinosteroid signals to promote seed germination in Arabidopsis

**DOI:** 10.1093/jxb/erab192

**Published:** 2021-05-08

**Authors:** Chunmei Zhong, Barunava Patra, Yi Tang, Xukun Li, Ling Yuan, Xiaojing Wang

**Affiliations:** 1 College of Forestry and Landscape Architecture, South China Agricultural University, Guangzhou, China; 2 Department of Plant and Soil Sciences, Kentucky Tobacco Research and Development Center, University of Kentucky, Lexington, KY, USA; 3 Guangdong Provincial Key Laboratory of Biotechnology for Plant Development, School of Life Science, South China Normal University, Guangzhou, China; 4 University of Birmingham, UK

**Keywords:** *Arabidopsis thaliana*, BEE2, bHLH, brassinosteroids (BRs), GASA6, gibberellins (GAs), HBI1, seed germination

## Abstract

Seed germination is regulated by multiple phytohormones, including gibberellins (GAs) and brassinosteroids (BRs); however, the molecular mechanism underlying GA and BR co-induced seed germination is not well elucidated. We demonstrated that BRs induce seed germination through promoting testa and endosperm rupture in Arabidopsis. BRs promote cell elongation, rather than cell division, at the hypocotyl–radicle transition region of the embryonic axis during endosperm rupture. Two key basic helix–loop–helix transcription factors in the BR signaling pathway, HBI1 and BEE2, are involved in the regulation of endosperm rupture. Expression of *HBI1* and *BEE2* was induced in response to BR and GA treatment. In addition, *HBI1*- or *BEE2*-overexpressing Arabidopsis plants are less sensitive to the BR biosynthesis inhibitor, brassinazole, and the GA biosynthesis inhibitor, paclobutrazol. HBI1 and BEE2 promote endosperm rupture and seed germination by directly regulating the *GA-Stimulated Arabidopsis 6* (*GASA6*) gene. Expression of *GASA6* was altered in Arabidopsis overexpressing *HBI1*, *BEE2*, or SRDX-repressor forms of the two transcription factors. In addition, HBI1 interacts with BEE2 to synergistically activate *GASA6* expression. Our findings define a new role for GASA6 in GA and BR signaling and reveal a regulatory module that controls GA and BR co-induced seed germination in Arabidopsis.

## Teaser

A transcriptional module amplifies the gibberellin and brassinosteroid signal to accelerate endosperm rupture to promote seed germination in Arabidopsis.

## Introduction

In plants, the freshly formed seeds maintain dormancy until the proper time of germination. Seed germination is a critical process in the plant life cycle and relies on networks of interconnected signal transduction pathways that integrate multiple hormonal and environmental signals (Bewley, 1997; Koornneef *et al.*, 2002; Bentsink and Koornneef, 2008; Seo *et al.*, 2009; Shu *et al.*, 2016; Topham *et al.*, 2017). Gibberellins (GAs) and brassinosteroids (BRs) promote seed germination, while abscisic acid (ABA) represses it (Finch-Savage and Leubner-Metzger, 2006; Bentsink and Koornneef, 2008; Weitbrecht *et al.*, 2011; Gallego-Bartolome *et al*., 2012; Tuan *et al.*, 2018; Kim *et al.*, 2019). A high GA/BR and low ABA level is a favorable condition for seed germination.

Multiple ABA-responsive transcription factors (TFs), including ABSCISIC ACID INSENSITIVE5 (ABI5), play key roles in inhibition of seed germination (Finkelstein and Lynch, 2000; Carles *et al.*, 2002; Lopez-Molina *et al.*, 2002; Skubacz *et al.*, 2016). ABI5 binds to the ABA-responsive element (ABRE) in the promoters of the genes encoding late embryogenesis abundant (LEA) proteins, such as *EARLY METHIONINE-LABELED 1* (*EM1*) and *EM6*, to repress their expression (Carles *et al.*, 2002). ABI5 also plays roles in integrating external signals and the crosstalk between several growth hormones, including GA and BR (Skubacz *et al.*, 2016).

GA promotes seed germination by directly inducing the expression of the genes involved in cell division and elongation or derepression of gene expression by degrading DELLA proteins, negative regulators of GA signaling. Degradation of DELLA proteins, particularly RGL2, also reduces ABA biosynthesis and promotes seed germination (Piskurewicz *et al.*, 2008). The antagonistic interaction between GA and ABA in controlling seed germination has been extensively studied (Bewley, 1997; Olszewski *et al.*, 2002; Finch-Savage and Leubner-Metzger, 2006; Piskurewicz *et al.*, 2008; Weitbrecht *et al.*, 2011; Liu *et al.*, 2016; Liu and Hou, 2018).

BR promotes seed germination by controlling the inhibitory effect of ABA on seed germination (Hu and Yu, 2014; Zhao *et al.*, 2019). Extensive physiological, biochemical, and genetic studies, mainly using Arabidopsis, have led to the identification and functional characterization of the components of BR signal transduction (Li *et al.*, 2001, 2002; Nam and Li, 2002; Tang *et al.*, 2011). BR signaling begins with the perception of the hormone ligand by the plasma membrane-associated receptor complex consisting of BRASSINOSTEROID-INSENSITIVE1 (BRI1) and BRI-ASSOCIATED KINASE1 (BAK1). The activated BRI1–BAK1 receptor complex phosphorylates BR-SIGNALING KINASE 1 (BSK1) and CONSTITUTIVE DIFFERENTIAL GROWTH 1 (CDG1), which further phosphorylates the PP1 type phosphatase BRI1 SUPPRESSOR 1 (BSU1). BSU1, along with PROTEIN PHOSPHATSE 2A (PP2A), dephosphorylates and inactivates the glycogen synthase kinase3-like kinase BRASSINOSTEROID INSENSITIVE2 (BIN2). Inactivation of BIN2 promotes the accumulation of positive regulators of BR signaling, BRASINAZONE-RESISTANT 1 (BZR1) and BRI1-EMS-SUPPRESSOR 1 (BES1), which directly control the transcription of BR-responsive genes to regulate plant developmental events (Li *et al.*, 2001; Kim *et al.*, 2011; Planas-Riverola *et al.*, 2019). Overexpression of *BZR1* diminishes the inhibitory effect of ABA in transgenic Arabidopsis plants (Tsugama *et al.*, 2013). In the absence of BR, BIN2 phosphorylates BZR1 and BES1 to repress their DNA binding capacity (He *et al.*, 2002; Wang *et al.*, 2002; Yin *et al.*, 2002; Ryu *et al.*, 2007). BES1 physically interacts with ABI5 to hinder its DNA binding capacity, attenuating the ABA-mediated suppression of seed germination by lowering the expression of ABI5 targets (Zhao *et al.*, 2019). BIN2 is a repressor of BR signal, but it promotes the ABA responses. During seed germination, BIN2 physically interacts with ABI5 to phosphorylate and stabilize AB15 in the presence of ABA. BIN2 and ABI5 mutually modulate the ABA-induced inhibition of seed germination. However, BRs antagonize the BIN2–ABI5 cascade and promote seed germination (Hu and Yu, 2014), indicating a complex hormonal crosstalk during seed germination.

Both BR and GA promote cell expansion and seed germination. The physical interaction of DELLA and BZR1 seems to be the molecular basis for the BR–GA crosstalk (Gallego-Bartolome *et al*., 2012; Ross and Quittenden, 2016; Ross *et al.*, 2016). BR can rescue the germination phenotypes of the GA biosynthetic mutant, *ga1-3,* and the GA-insensitive mutant, *sleepy1* (Steber and McCourt, 2001), suggesting that BR-induced seed germination does not totally depend on GA response, but rather the hormones working in parallel. Seeds of both the BR biosynthetic mutant *det2-1* and the BR-insensitive mutant *bril1-1* are able to germinate without BR and are hypersensitive to ABA. In rice, seed germination and seedling growth are significantly affected by the BR biosynthetic inhibitor brassinazole (BRZ) which is completely recovered by treatment with GA (Li *et al.*, 2020). These observations indicate that BRs play an auxiliary role in the GA-promoted regulation of seed germination and can reverse the inhibitory effect of ABA (Steber and McCourt, 2001). A recent study using iTRAQ (isobaric tag for relative and absolute quantification) proteomic analysis has revealed that GAs and BRs coordinately regulate rice seed germination and embryo development by modulating the expression of several common targets (Li *et al.*, 2018). However, the molecular mechanisms underlying GA- and BR-induced seed germination have not been thoroughly investigated.

The interacting transcriptional module, DELLA/BZR1/PHYTOCHROME INTERACTING FACTOR4 (PIF4), integrates GA, BR, and light signals to mediate cell elongation (Bai *et al.*, 2012b; Li *et al.*, 2012; Oh *et al.*, 2012). Under low GA conditions, DELLA interacts with BZR1 and PIF4 to inhibit their DNA binding activity, thus inhibiting cell growth and elongation. The promotion of cell elongation by BZR1–PIF4 requires a tripartite helix–loop–helix/basic helix–loop–helix (HLH/bHLH) module that consists of PACLOBUTRAZOL-RESISTANT (PRE), ILI1 BINDING bHLH PROTEIN1 (IBH1), and HOMOLOG OF BEE2 INTERACTING WITH IBH1 (HBI1) (Bai *et al.*, 2012a; Fan *et al.*, 2014; Zheng *et al.*, 2019). HBI1 is also a positive regulator of BR signaling and functionally redundant with another bHLH TF, BEE2 (BRASSINOSTEROID ENHANCED EXPRESSION2) (Malinovsky *et al.*, 2014). Genetic evidence suggests that HBI1 plays a pivotal role in GA-induced cell elongation. A total of 177 direct targets of HBI1 have been identified by chromatin immunoprecipitation sequencing (ChIP-Seq) and RNA sequencing (RNA-Seq), several of which encode cell wall-related proteins, such as expansins (*EXP2*) and GA-stimulated Arabidopsis (GASA) family proteins (*GASA4* and *GASA6*) (Rubinovich and Weiss, 2010; Bai *et al.*, 2012a; Fan *et al.*, 2014; Yan *et al.*, 2014; Zhong *et al.*, 2015). In Arabidopsis, the GASA family is represented by 14 members, of which GASA4 and GASA6 are positive regulators of GA response ( Zhang and Wang, 2008; Rubinovich and Weiss, 2010; Zhong *et al.*, 2015). Our previous study suggests that GASA6 regulates seed germination by serving as an integrator for the GA, ABA, and glucose (Glc) signaling cascades (Zhong *et al.*, 2015).

In this study, we demonstrate that, similarly to GA, BRs also promote seed germination by accelerating endosperm rupture through promoting cell elongation at the hypocotyl–radicle transition region. In addition, we provide genetic and molecular evidence that two GA- and BR-responsive bHLH TFs, HBI1 and BEE2, directly bind to E box elements in the *GASA6* promoter to regulate its expression. We illustrate a mechanism in which a bHLH TF complex mediates GA/BR-induced seed germination through activation of *GASA6.*

## Materials and methods

### Plant material and growth conditions

All mutant and transgenic lines were in the *Arabidopsis thaliana* accession Col-0. Seeds were surface-sterilized and sown on plates with half-strength basal Murashige and Skoog (MS) medium (Sigma-Aldrich, USA) containing 0.8% (w/v) agar (MBCHEM, China). Plants were grown in a climate-controlled room (22 °C, photoperiod of 16 h light/8 h dark, light intensity of ~100 µmol m^−2^ s^−1^, and relative humidity of 70%). *HBI1-OE* (overexpressing) and *BEE2-OE* lines were kindly provided by Dr Cyril Zipfel (Sainsbury laboratory, Norwich, UK), and *HBI1-SRDX* and *BEE2-SRDX* lines were kindly provided by Dr Masaru Ohme-Takagi (Bioproduction research institute, Tsukuba, Japan). *HBI1-OE/gasa6* and *BEE2-OE/gasa6* were generated by a genetic cross between *gasa6* (SALK_072904) and *HBI1-OE* or *BEE2-OE*, and homozygous lines were verified by PCR using the primers listed in Supplementary Table S1.

### Germination assay and hypocotyl length assay

For each germination assay, three independently grown seed batches of the wild type (WT), *HBI1-OE*, or *BEE2-OE* were compared. To ensure synchronous germination, seeds were imbibed at 4 °C for 3 d, then moved to a growth chamber with a 16 h/8 h light/dark cycle at 22 °C. The experiments were performed on half-strength MS medium supplemented with 1 µM 2,4-epibrassinolide (BR), 1 µM BRZ (Sigma-Aldrich, USA), 100 µM gibberellin (GA_3_), 1 µM paclobutrazol (PAC) (Sigma-Aldrich, USA), or 100 µM ABA (Sigma-Aldrich, USA). At least 80 seeds were imbibed for each treatment and examined for testa and endosperm rupture under a SMZ1500 stereomicroscope (Nikon, Japan), and photographed with a high-resolution digital camera (COOLPIX4500, Nikon, Japan). Germination rate was determined by calculating the percentage of testa and endosperm rupture in the control and different treatments. In the hypocotyl length assay, seeds were incubated for 36 h to attain 100% germination because BR-treated seeds germinate faster than those of the WT. After germination, testas were stripped and 50–70 embryos were photographed with a BX51 camera (Olympus, Japan). Hypocotyl length was measured using the Image J software (https://imagej.nih.gov/ij/index.html). The SPSS software (http://www.spss.com/) was used for statistical analysis throughout this study.

### Measurement of embryonic axis epidermal cells

To ensure the synchronous and full germination of both untreated and treated seeds, we incubated the seeds for 36 h at room temperature before taking the measurements. Seeds were collected at 36 h and fixed in 50% (v/v) methanol and 10% (v/v) acetic acid overnight at 4 °C. Embryos were dissected from testas and stained as described previously (Sliwinska *et al.*, 2009), and subsequently photographed with an LSM510 Meta confocal laser-scanning microscope (Zeiss, Germany). Photographs were enlarged electronically for measurement of cell length and width with Image J software.

### Gene expression analysis

Quantitative real-time PCR (qRT-PCR) was performed as previously described (Zhong *et al.* 2015). Briefly, total RNA was extracted from 2-week-old seedlings or seeds using the total RNA isolation Kit (Promega, USA) according to the manufacturer’s instruction. About 800 ng of total RNA for each sample was reverse transcribed using the PrimeScript RT Reagent Kit with gDNA Eraser (TAKARA, Japan). All PCRs were performed using SYBR Premix Ex Taq Mix (TAKARA, Japan) in triplicate and repeated at least three times. The transcript levels were measured by the comparative cycle threshold (Ct) method (bulletin no. 2; Applied Biosystems, http://www.appliedbiosystems.com). *Ubiquitin1* (*UBQ1*) (Jiang *et al.*, 2012) and *Tubulin 3* (*TUB3*) (Patra *et al.*, 2013) were used as internal controls. Primers used for qRT-PCR are listed in Supplementary Table S1. β-Glucuronidase (GUS) assay was performed as previously described (Zhong *et al.*, 2015). Briefly, T_3_ transgenic lines carrying different truncated *GASA6* promoters fused with the *GUS* reporter gene were analyzed for GUS histochemical staining. Samples were incubated in the GUS staining solution [1 mg ml^–1^ 5-bromo-4-chloro-3-indolyl glucuronide (X-Gluc) dissolved in 50 mM Na-phosphate buffer] at 37 °C overnight, then bleached using 70% (v/v) ethanol. All the samples were photographed under an Olympus BX Microscope (Olympus, Japan).

### Yeast two-hybrid assay

The cDNA encoding either the full length or fragments of the desired proteins were fused to pGADT7 [activation domain (AD)] or pGBKT7 [DNA-binding domain (BD)]. The AD and BD fusion plasmids were paired in different combinations and co-transformed into *Saccharomyces cerevisiae* strain AH109 (Clontech, USA). Transformed colonies were then selected on synthetic dropout (SD) medium lacking leucine and tryptophan (–Leu –Trp). Interactions were determined by growth of the colonies on SD medium lacking histidine, leucine, and tryptophan (–His –Leu –Trp), and containing 5 mM (for HBI1) 3-amino-1,2,4-triazole (3-AT). Primers used for plasmid construction for yeast two-hybrid assay are listed in Supplementary Table S1.

### Bimolecular fluorescence complementation (BiFC) assay

Full-length *HBI1*, *BEE2*, or *IBH1* cDNAs were cloned into the pSAT6-nYFPC1 or pSAT6-cYFPC1 vectors, which contained either the N- or the C-terminal half of yellow fluorescent protein (YFP). The resulting constructs were paired in different combinations and co-transformed into the *A. thaliana* mesophyll protoplasts as described previously (Yoo *et al.*, 2007). The YFP signals were observed with an LSM510 Meta confocal laser-scanning microscope (Zeiss, Germany). Primers used for plasmid construction for the BiFC assay are listed in Supplementary Table S1.

### Protoplast transient assay

Different fragments of the *GASA6* (1.4, 1.2, 1.1, or 0.9 kb) promoter were each cloned into the *pGreen II 0800-LUC* vector (Hellens *et al.*, 2005) to generate reporter constructs. Full-length *HBI1*, *BEE2*, or *IBH1* cDNAs were cloned into the pBlueScript vector with the *Cauliflower mosaic virus* (*CaMV*) 35S promoter and *rbcS* terminator to generate effector constructs. Each reporter construct, together with either *35S::HBI1*, *35S::BEE2*, or *35S::IBH1*, was co-transformed into the mesophyll protoplasts of *A. thaliana* for transcriptional activity assay.

Single or double mutants of the *GASA6* (1.4 kb) promoter were generated with the MutanBEST Kit (TAKARA, Japan) and subsequently cloned into the *pGreen II 0800- LUC* vector. Firefly and Renilla luciferase activities were assayed with the microplate luminometer (Turner Biosystems, USA) and the Dual-Luciferase Reporter Assay reagents (Promega, USA). Primers used for plasmid construction for protoplast assay are listed in Supplementary Table S1.

### ChIP-PCR assay

ChIP assays were performed as previously described (Hou *et al.*, 2014). Briefly, 5-day-old seedlings of *35S::HBI1-YFP-HA* or *35S::BEE2-YFP-HA* were fixed on ice for 45 min in 1% formaldehyde under vacuum. Fixed tissues were homogenized, and chromatin was isolated and sonicated to generate DNA fragments with an average size of 500 bp. The solubilized chromatins were immunoprecipitated by Protein A+G magnetic beads (Magna, USA) with anti-HA (Sigma-Aldrich, USA), and the co-immunoprecipitated DNAs were subsequently recovered and analyzed by qPCR with the SYBR Premix Ex Taq Mix (TAKARA, Japan). The relative fold enrichment was calculated by normalizing the amount of target DNA fragments against the respective input DNA samples and then against the amount of *PP2A* genomic fragments. Primers used for ChIP-PCR are listed in Supplementary Table S1.

### Recombinant protein production in bacteria, and EMSA

To produce recombinant HBI1 and BEE2 proteins in bacteria, the corresponding ORFs were cloned into the pGEX4T1 vector (GE Healthcare Biosciences, USA). The resulting plasmids were transformed into BL21 cells containing pRIL (Agilent, USA). Protein expression was induced by adding 0.2 mM isopropyl-β-d-thiogalactopyranoside (IPTG) to the cell cultures at *A*_600_ ~1.0 and incubated for 3 h at 37 °C. The cells were harvested and lysed using CelLytic B (Sigma-Aldrich, USA). The glutathione *S*-transferase (GST) fusion proteins were bound to glutathione–Sepharose 4B columns (Amersham, USA) and then eluted by 10 mM reduced glutathione in 50 mM Tris–HCl buffer (pH 8.0) (Patra *et al.*, 2018).

For EMSA, two (GASA1 37 bp and GASA2 30 bp) probes were synthesized, with or without 5′ biotin labeling (Integrated DNA Technology, USA). The probes were designed to include the potential binding sites, as indicated by the ChIP experiment and protoplast transient assay. Oligos were annealed to produce double-stranded probes for EMSA using nuclease-free duplex buffer (Integrated DNA Technology, USA). The DNA-binding reactions were carried out in 10 mM Tris, pH 7.5, 50 mM KCl, 5 mM MgCl_2_, 1 mM DTT, 2.5% glycerol, 0.5% NP-40, and 50 ng of poly(dI–dC) in a final volume of 20 µl. Purified proteins were incubated with 25 fmol DNA probe at room temperature for 45 min. For the competition experiment, cold probes were added in an excess molar ratio (1000 times). The DNA–protein complexes were resolved by electrophoresis on 6% non-denaturing polyacrylamide gels and then transferred to BiodyneB modified membrane (0.45 mm; Pierce, USA). The band shifts were detected by a chemiluminescent nucleic acid detection module (Pierce, USA) and exposed to X-ray films.

## Results

### BRs promote seed germination by accelerating cell expansion

**Fig. 1.**
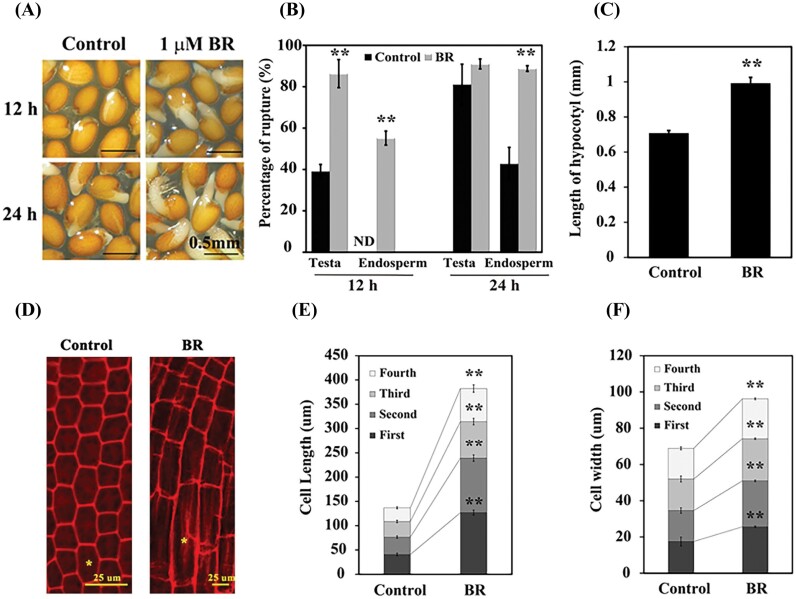
BR accelerates seed germination by promoting cell expansion. (A) Germination phenotypes of wild-type (WT) Arabidopsis (Col) seeds treated with 1 µM BR for 12 h and 24 h. (B) Percentages of testa and endosperm rupture in (A). (C) Hypocotyl length of WT seeds treated with 1 µM BR at 36 h. (D) Images of embryonic axis cells of WT seeds treated with 1 µM BR at 36 h; the yellow asterisk indicates the first cell. (E and F) Cell length and width, respectively, of the embryonic axis of WT seeds in (D). Seeds germinated on half-strength basal MS were used as control. ND, no detection. The black asterisks indicate significant differences compared with control (one-way ANOVA was used to analyze the significant differences). Three biological replicates were used for analysis. *P<0.05; **P<0.01.

The completion of seed germination requires two sequential steps: shortly after imbibition, the testa and endosperm rupture consecutively, followed by radicle emergence (Bentsink and Koornneef, 2008; Weitbrecht *et al.*, 2011). We analyzed the Arabidopsis seed germination in the presence of 2,4-epibrassinolide (one of the biologically active brassinosteroids, hereafter referred to as BR). Seeds were germinated on half-strength MS medium alone or supplemented with 1 µM BR. At 12 h, ~40% of testa rupture was observed in control seeds, compared with 86% in BR-treated seeds. Endosperm rupture was significantly higher (55%) in BR-treated seeds compared with the control (undetectable). Similar patterns of endosperm rupture were also observed at 24 h (Fig. 1A, B), suggesting that BR promotes seed germination by accelerating testa and endosperm rupture.

Hypocotyl elongation assays were conducted under similar conditions to those of germination assays. BR significantly promoted the hypocotyl length by ~30% compared with the control (Fig. 1C). The sizes of the four cells in the hypocotyl–radicle transition region were measured to determine whether the effects of BR on embryo axis growth were caused by cell division or cell elongation. The cell lengths were greater in the presence of BR compared with the control (Fig. 1D, E). The effects of BR on the four cells in the transition region seem to be gradual, as in the presence of BR the lengths of the cell closest to the radicle (the first cells) increased 86 µm in contrast to the fourth cells which increased 40 µm (Fig. 1D, E). A similar effect of BR was observed on cell width (Fig. 1D, F). These observations imply that BR accelerates endosperm rupture by promoting cell elongation in the hypocotyl–radicle transition region of the embryo.

### HBI1 and BEE2 mediate endosperm rupture mainly via enhancing BR and GA responses

**Fig. 2.**
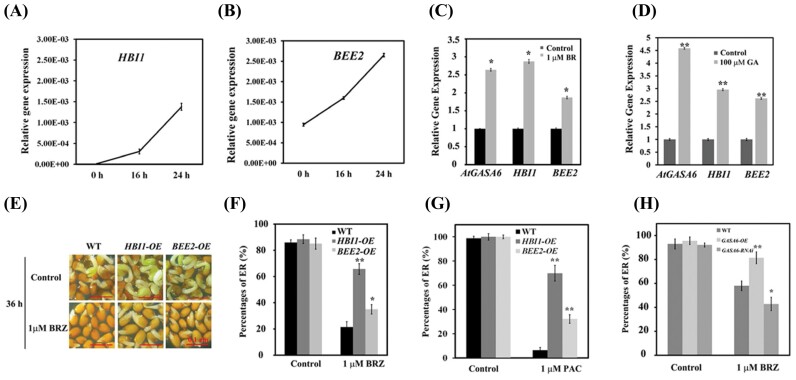
*HBI1* and *BEE2* are involved in BR-mediated endosperm rupture. Relative transcript levels of *GASA6*, *HBI1*, and *BEE2* in response to BR and GA_3_ as measured using quantitative RT-PCR (qRT-PCR). The expression of *HBI1* (A) and *BEE2* (B) during the course of seed germination; 0 h was marked as the time point when seeds were exposed to light after 3 d of stratification. Two-week-old seedlings were treated with 1 µM BR (C) or 100 µM GA_3_ (D) for 2 h. The transcript levels were normalized to *UBQ1*. Data represent the mean ±SE (*n*=3). One-way ANOVA was used to analyze any significant difference. All experiments were repeated at least twice with similar results. (E) Images of germination phenotypes of WT (Col), HBI1*-OE*, or BEE2*-OE* seeds treated with 1 µM BRZ for 36 h; scale bar=0.1 cm. (F) Percentages of endosperm rupture (ER) in WT, *HBI1-OE*, or BEE2*-OE* seeds in (E). (G) Percentages of ER of WT, HBI1*-OE*, or BEE2*-OE* seeds treated with 1 µM PAC for 54 h. (H) Percentage of ER in WT, *GASA6-OE* and *GASA6-RNAi* seeds treated with 1 µM BRZ at 48 h. Seeds germinated on half-strength basal MS was used as the control. The asterisks indicate significant differences compared with control or the WT (one-way ANOVA was used to analyze significant differences). Three biological replicates were used for analysis. *P<0.05; **P<0.01.

HBI1 and its closest homolog BEE2 are known to act downstream of BR and GA signaling pathways to promote cell elongation (Bai *et al.*, 2012a). However, their roles in seed germination have not been investigated. We performed qRT-PCR to examine the expression of *HBI1* and *BEE2* during Arabidopsis seed germination as well as their response to BR and GA treatments. The seeds were stratified for 3 d, and expression of *HBI1* and *BEE2* was measured at 0, 16, and 24 h of light exposure. The expression of both *HBI1* and *BEE2* increased gradually during seed germination. Transcript abundance of BEE2 was higher than that of HBI1 in germinating seeds (Fig. 2A, B; Supplementary Fig. S1). Consistent with previous observations, the expression of both regulators was significantly induced following BR or GA treatment (Fig. 2C, D; Supplementary Fig. S2A, B). Next, we determined whether ABA regulates expression of HBI1 and BEE2 to influence endosperm rupture. We found that the expression of HBI1 and BEE2 was significantly increased in the *abi5* mutant compared with that in the WT (Supplementary Fig. S3). Additionally, ABA repressed the expression of HBI1 in WT seeds while BEE2 expression did not change significantly (Supplementary Fig. S4). Next, we compared endosperm rupture in seeds of HBI1 and BEE2 overexpression lines (*HBI1-OE* and *BEE2-OE*) with that of the control (WT) in the presence of the BR biosynthesis inhibitor, BRZ. Compared with the WT, the *HBI1-OE* and *BEE2-OE* seeds were less sensitive to BRZ, as evidenced by significantly higher percentages of endosperm rupture in the BRZ-supplemented medium (Fig. 2E, F). In addition, we compared the percentage of edosperm rupture of *HBI1-OE* and *BEE2-OE* with that of the WT in the presence of the GA biosynthesis inhibitor, PAC. Under the control condition, *HBI1-OE* showed no difference in seed germination compared with the WT, but in the presence of PAC, *HBI1-OE* showed an ~65% increase of seed germination (Fig. 2G). Similarly, the percentage of endosperm rupture of *BEE2-OE* was significantly higher than that of the WT in the presence of PAC (Fig. 2G). In addition, overexpression of HBI1 (*HBI1-OE*) diminished the negative effect of ABA on seed germination, as evident by the higher percentage of endosperm rupture compared with that of WT and *BEE2-OE* seeds when treated with ABA (Supplementary Fig. S5A). These findings suggest that overexpression of HBI1 enhances the BR and GA responses in regulating endosperm rupture, and HBI1 is capable of counteracting the inhibitory effect of ABA during seed germination.

### HBI1 and BEE2 promote seed germination, probably via GASA6

HBI1 is a potential regulator of genes encoding many cell wall-related proteins, such as expansins and GASAs (Fan *et al.*, 2014). A previous study has shown that *GASA6* acts as a positive regulator in GA-, ABA-, and Glc-mediated seed germination (Zhong *et al.*, 2015). However, the involvement of *GASA6* in BR signaling is not well studied. We performed qRT-PCR to determine the effect of BR on *GASA6* expression. Similar to *HBI1* and *BEE2*, *GASA6* expression was significantly activated by BR and GA (Fig. 2C, D; Supplementary Fig. S2A, B). Also similar to *HBI1*, expression of *GASA6* was induced in *abi5* seeds and reduced in WT seeds in the presence of ABA (Supplementary Figs S3A, B, S4A, B). In addition, the seed germination efficiencies of *GASA6*-overexpressing lines (*GASA6-OE*) and RNAi lines (*GASA6-RNAi*) were evaluated in the presence of BRZ. In the control conditions, no significant change in seed germination was observed for either *GASA6-OE* or RNAi lines (Fig. 2H). However, in the presence of 1 µM BRZ, seeds of *GASA6-OE* showed increased germination compared with the WT, whereas seeds of *GASA6-RNAi* displayed decreased germination (Fig. 2H). Similarly, in the presence of ABA, *GASA6-OE* seeds showed a significantly improved germination rate compared with the WT and *GASA6-RNAi* (Supplementary Fig. S5B). These results suggest that HBI1 and GASA6 act coordinately to promote BR- and GA-mediated seed germination and attenuate the negative effect of ABA during seed germination.

### HBI1 and BEE2 directly regulate *GASA6* expression in Arabidopsis

**Fig. 3.**
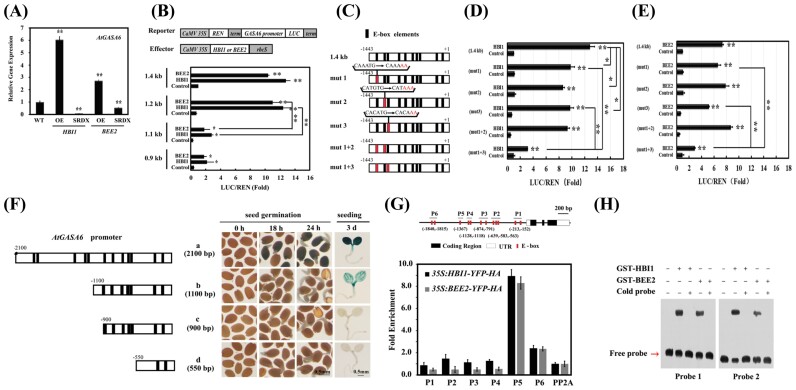
HBI1 and BEE2 regulate *GASA6* expression by binding to the E-box-like elements *in vivo* and *in vitro.* (A) Transcript levels of *GASA6* in *HBI1-* or *BEE2-OE* and SRDX lines measured using quantitative RT-PCR (qRT-PCR). Data were normalized to *UBQ1*. (B) Transactivation of the full*-*length and truncated *GASA6* promoter–reporters by HBI1 or BEE2 in Arabidopsis protoplasts. Various constructs used in transient expression assays are shown in the upper panel. (C) Schematic diagram of the 1.4 kb *GASA6* promoter with all E-boxes (black) and mutated E-boxes (red) used in (D) and (E). Transactivation of the *GASA6* promoter and its mutants by HBI1 (D) or BEE2 (E) in Arabidopsis protoplasts. (F) Schematic diagram of different fragments of the *GASA6* promoter (left); the numbers indicate the promoter length. Analysis of GUS activities in different *pGASA6::GUS* lines (right). Scale bar=0.5 mm. (G) Schematic diagram of the *GASA6* promoter. P1 to P6 indicate fragments used for chromatin immunoprecipitation-quantitative PCR (ChIP-qPCR) amplification. ChIP-qPCR analysis of HBI1-HA or BEE2-HA binding to the *GASA6* promoter upon precipitation with anti-HA antibody. Five-day-old *35S::HBI1-YFP-HA*, *35S::BEE2-YFP-HA*, or WT seedlings were used in ChIP-qPCR. Fold enrichments indicate the enrichment of HBI1-HA or BEE2-HA binding to the *GASA6* promoter compared with that of the WT. Data represent the mean ±SE of three replicates. (H) EMSA of HBI1 or BEE2 after incubation with biotin-labeled DNA probes containing the E-box sequences of the *GASA6* promoter (Probe1 and Probe2). In competition experiments to demonstrate the specific binding of proteins to the probes, non-labeled probes (cold probes) were added in 1000-fold excess of the labeled probes. Values represent the mean ±SE of at least four biological replicates. Except when specifically indicated, asterisks indicate significant differences compared with control (one-way ANOVA was used to analyze the significant differences). *P<0.05; **P<0.01.

*In silico* analysis identified a total of 12 bHLH TF-binding motifs (E-box elements) in the *GASA6* promoter (Supplementary Fig. S6). In addition, the co-expression analysis using the ATTED-II network drawer revealed that both *HBI1* and *BEE2* are co-expressed with *GASA6* (Supplementary Figs S7–S9). To test whether HBI1 and BEE2 regulate *GASA6* expression through binding to the E-box elements, we first determined *GASA6* expression in the *HBI1-OE* and *BEE2-OE* lines, as well as in the *HBI1-SRDX* and *BEE2-SRDX* lines. As HBI1 and BEE2 are functionally redundant, it is assumed that the single knockout mutants will not show an obvious phenotype (Malinovsky *et al.*, 2014). Therefore, we used the *HBI1-SRDX* and *BEE2-SRDX* lines, in which the dominant repressor form of HBI1 or BEE2 specifically suppresses the target genes, thus preventing the possible interference of functional redundancy (Hiratsu *et al.*, 2003; Ikeda *et al.*, 2012). The results showed that *GASA6* expression was significantly higher in *HBI1-OE* or *BEE2-OE* lines than that in the WT but was repressed markedly in the *HBI1-SRDX* or *BEE2-SRDX* lines (Fig. 3A; Supplementary Fig. S10), indicating that HBI1 and BEE2 regulate the expression of *GASA6* in Arabidopsis.

To identify the binding sites of HBI1 and BEE2 in the *GASA6* promoter, we performed transient expression assays using Arabidopsis protoplasts. Truncated fragments of the *GASA6* promoter, cloned into the pGreenII 0800-LUC vector (Hellens *et al.*, 2005), served as reporters. *HBI1* or *BEE2*, expressed under the control of the *CaMV* 35S promoter, were used as effectors. The reporters and effectors were co-transformed into Arabidopsis mesophyll protoplasts in different combinations. HBI1 or BEE2 significantly induced the activities of the 1.4 kb and 1.2 kb *GASA6* promoters (upstream of the ATG start codon). However, their effects were dramatically reduced on the 1.1 kb and 0.9 kb promoter (Fig. 3B). These observations indicate that the 100 bp region between 1.2 kb and 1.1 kb of the *GASA6* promoter is likely to be critical for the HBI1- and BEE2-induced expression. We identified three E-box elements (CAAATG, CATGTG, and CACATG) between 1.4 kb and 1.1 kb. To clarify the importance of these three E-box elements in HBI1/BEE2-controlled expression, point mutants were generated individually in the three E-boxes and used in the Arabidopsis transient expression assays. As shown in Fig. 3C, the last two nucleotides (TG) in all three E-box elements were replaced with AA to generate mutant promoters, mut 1, 2, 3, and their combination, mut 1 + 2 and mut 1 + 3 (Fig. 3C). The transient expression assays showed that, compared with the activation of the WT promoter, the activation of the three single mutant promoters by HBI1 was significantly reduced, whereas only mut 3 affected the activation by BEE2. However, the double mutation, mut1 + 3, significantly reduced the activation by either HBI1 or BEE2 (*P*<0.001) (Fig. 3D, E), indicating that two or more distantly located E-boxes are required for the regulation of *GASA6* by the two factors.

To further validate this, we performed 5′ end deletion analysis of the *GASA6* promoter using transgenic plants expressing various truncated promoter fragments fused to the *GUS* reporter gene. Analysis of the transgenic plants revealed that the region between –2100 bp and –1100 bp, where multiple E-boxes reside, is potentially important for *GASA6* expression during seed germination and seedling growth (Fig. 3F).

Next, we performed ChIP-PCR assay using transgenic plants expressing *HBI1-YFP-HA* or *BEE2-YFP-HA* to measure enrichment of the E-boxes in the *GASA6* promoter. The WT plants served as control. HBI1 strongly bound to the E-box-containing regions of P5 (–1367 bp upstream of ATG) in the *GASA6* promoter (Fig. 3G). A similar DNA enrichment pattern by the BEE2-YFP-HA protein was also detected in the *GASA6* promoter (Fig. 3G), suggesting that HBI1 and BEE2 co-target the *GASA6* promoter, probably via direct binding to the E-box elements. However, the E-box elements (CACATG and CATGTG) in the 100 bp region are different from the one (CAAATG) in the P5 fragment, suggesting that the mechanisms of HBI1 or BEE2 regulating *GASA6* expression are somewhat intricate. Direct binding of HBI1 or BEE2 to the E-box elements in the *GASA6* promoter was further verified by EMSA, where two DNA probes, with or without 5′ biotin labeling, were synthesized based on the E-box elements of the *GASA6* promoter (Probe 1, caatttttaatca***caaatg***ctattttattggacgacc; Probe 2, GTCTCC***CATGTG***AGTG***CACATG***GAGTTATG). The results showed that both HBI1 and BEE2 individually bind to the E-box elements, resulting in a mobility shift. The specificity of the DNA–protein interaction was confirmed by the competition experiment, in which the addition of excess (1000×) non-labeled probe eliminated the interaction between labeled probe and HBI1 or BEE2 (Fig. 3H).

### BEE2 and HBI1 form a heterodimer and synergistically regulate expression of *GASA6*

**Fig. 4.**
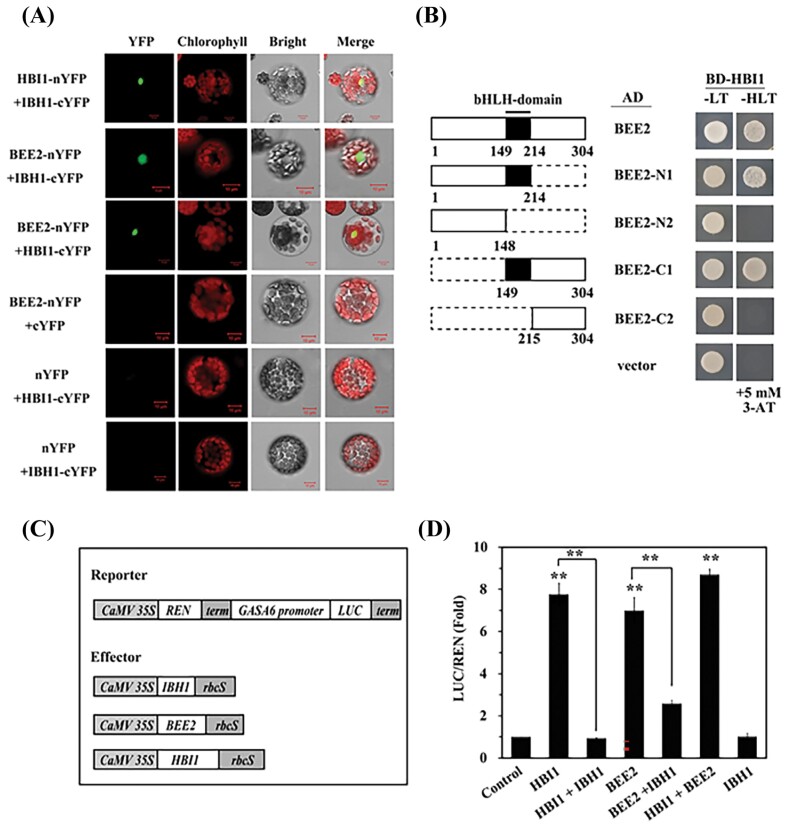
HBI1 and BEE2 modulate the expression of *GASA6* by forming a homodimer or heterodimer *in vivo*. (A) Bimolecular fluorescent complementation (BiFC) assay shows that HBI1 and BEE2 interact with each other and IBH1 to form heterodimers in Arabidopsis mesophyll protoplasts. YFP, signal of yellow fluorescence protein; Chlorophyll, autofluorescence of chloroplasts; Bright, protoplasts in light view; Merge, merge of YFP, chlorophyll, and light view. (B) Diagram of the BEE2 domain structures and various deletions (left); yeast two-hybrid assays show the heterodimer of HBI1, and the interaction domain. Protein–protein interactions were detected by yeast growth on triple (–His–Leu–Trp) dropout selection medium, with 5 mM 3-amino-1,2,4-triazole (3-AT) (right). (C) Schematic diagram of various constructs used in transient expression assays (D). (D) Transactivation of the *GASA6* promoter (1.4 kb) by HBI1, BEE2, and IBH1 in Arabidopsis mesophyll protoplasts. The *GASA6* promoter fused to the LUC reporter was co-transformed with effectors or empty vector (control) into mesophyll protoplasts. Fold of LUC/REN indicates the expression level of *GASA6* activation by various effectors. Values represent the mean ±SE of four biological replicates. Except when specifically indicated, asterisks indicate significant differences compared with control (one-way ANOVA was used to analyze the significant differences). **P<0.01.

It has been well demonstrated that bHLH factors homo- or heterodimerize through the bHLH domain (Heim *et al.*, 2003; Toledo-Ortiz *et al.*, 2003). To determine whether HBI1 and BEE2 form a homo- or heterodimer, we first performed BiFC in Arabidopsis mesophyll protoplasts. Reciprocal fusions of BEE2, IBH1, and HBI1 with the N- or C-terminal half of YFP (nYFP and cYFP, respectively) were generated and co-transformed into the protoplasts in combinations. IBH1, a known interactor of BEE2 and HBI1, served as a positive control. Strong YFP fluorescence signals were observed in the nucleus when HBI1–nYFP or BEE2–nYFP was co-transformed with IBH1–cYFP and BEE2–nYFP was co-transformed with HBI1–cYFP, indicating specific interactions of HBI1–IBH1, BEE2–IBH1, and BEE2–HBI1 (Fig. 4A). Next, we performed yeast two-hybrid assays to confirm that the HBI1–BEE2 interaction is mediated by the bHLH domains. BEE2 was truncated into four fragments, with or without the bHLH domain, fused with the GAL4 AD, and co-expressed with HBI1 fused with the GAL4 BD (Fig. 4B. Only the bHLH domain-containing BEE2 fragments interacted with HBI1, confirming the requirement of the bHLH domain in dimerization.

To determine the biological significance of the dimerization among HBI1, BEE2, and IBH1, we performed transient expression assays in the protoplasts with different combinations of these effector proteins (Fig. 4C) on the *GASA6* promoter (p*GASA6-luc*). As shown in Fig. 4D, activation of the *GASA6* promoter was significantly higher when HBI1 and BEE2 were co-expressed (HBI1+BEE2) compared with HBI1 or BEE2 expressed alone (Fig. 4D). On the other hand, the addition of IBH1 significantly attenuated the activity of HBI1 or BEE2 (Fig. 4D), possibly by forming a non-DNA-binding complex with HBI1 or BEE2 as described previously (Ikeda *et al.*, 2012; Zhiponova *et al.*, 2014)

### GASA6 acts downstream of HBI1 and BEE2 to promote cell elongation

**Fig. 5.**
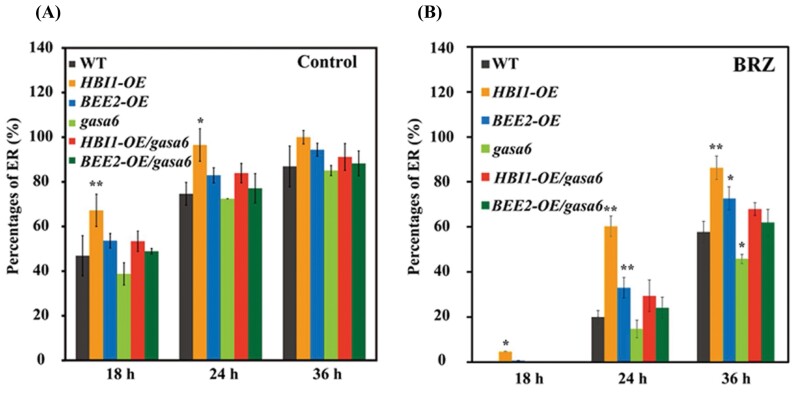
GASA6 acts downstream of HBI1 and BEE2 to promote seed germination. Endosperm rupture (ER) percentage of WT, *HBI1-OE*, *BEE2-OE*, *gasa6*, *HBI1-OE/gasa6* and *BEE2-OE/gasa6* seeds after 18, 24, and 36 h of germination (A) control (half-strength basal MS medium) and (B) half-strength basal MS medium with 1 mM BRZ. The black asterisks indicate significant differences compared with the WT (one-way ANOVA was used to analyze the significant differences). *P<0.05; **P<0.01.

To investigate the relationship between HBI1 or BEE2 and GASA6 at the genetic level, *HBI1-*OE*/gasa6* and *BEE2/gasa6* plants were generated by making a genetic cross between *HBI1-OE* or *BEE2-OE* transgenic plants and the homozygous *GASA6* T-DNA insertion mutant (*gasa6*)*. HBI1-OE* or *BEE2-OE* lines exhibited a BRZ-resistant phenotype. Although no significant difference was observed between the WT and *gasa6* under the control condition (Fig. 5A), the endosperm rupture percentage of *gasa6* was markedly reduced compared with the WT under 1 µM BRZ treatment (Fig. 5B). Furthermore, *HBI1-OE* or *BEE2-OE* in the *gasa6* background showed increased sensitivity to BRZ (~60%; Fig. 5B), compared with that without BRZ treatment (~90%; Fig. 5A), suggesting that, in the BR signaling cascade, GASA6 acts downstream of HBI1 and BEE2 to promote cell elongation.

## Discussion

BR and GA are principal plant growth regulators that function redundantly to control many important physiological functions, including seed germinations and cell elongation (Steber and McCourt, 2001; Hu and Yu, 2014; Li *et al.*, 2018; Zhao *et al.*, 2019). Physical interactions between BZR1/BES1 and DELLAs mediate the crosstalk between BRs and GAs during cell elongation in Arabidopsis (Bai *et al.*, 2012b; Gallego-Bartolome *et al*., 2012; Li *et al.*, 2012); however, the molecular mechanism underlying GA–BR crosstalk during seed germination is not well studied. In this study, we demonstrated that BR accelerates endosperm rupture by enhancing the growth of the hypocotyl–radicle transition region of the embryo (Fig. 1), similar to what has been observed previously in *GASA6* overexpression (Zhong *et al.*, 2015). The elongation of the embryonic axis in a completely germinated Arabidopsis seed is a result of cell elongation rather than cell division (Sliwinska *et al.*, 2009). As expected, the length and width of the cells in the hypocotyl–radicle transition region are significantly increased in the presence of BR (Fig. 1D–F), suggesting that BR affects cell elongation and width during embryonic axis elongation before endosperm rupture in Arabidopsis. Although the underlying mechanism requires further investigation, our findings provide a new insight into the coherent events at BR-promoted cell elongation during seed germination.

BZR1 and BES1 contribute to regulation of seed germination (Ryu *et al.*, 2014; Zhao *et al.*, 2019). The gain-of-function mutant *bes1-D* exhibits reduced sensitivity to ABA during seed germination, a phenotype not observed in the *bzr1-D* mutant, suggesting that BES1, but not BZR1, is the major contributor to BR-mediated suppression of ABA signaling during seed germination (Ryu *et al.*, 2014). In addition, BES1 physically interacts with ABI5 to attenuate the ABA-mediated suppression of seed germination by lowering the expression of ABI5 targets (Zhao *et al.*, 2019). Similar to that of *GASA6* (Zhong *et al.*, 2015), we found that *HBI1* and *BEE2* expression increased gradually during seed germination (Fig. 2A, B; Supplementary Fig. S1) and increased significantly in *abi5* mutant seeds (Supplementary Fig. S3). ABA repressed the expression of *HBI1* in WT seeds, while *BEE2* expression remained unchanged (Supplementary Fig. S4). We thus hypothesized that HBI1 and BEE2 are involved in regulation of seed germination. Supporting this notion is that the endosperm rupture of the *HBI1-OE* or *BEE2-OE* lines showed decreased sensitivity to the BR biosynthesis inhibitor BRZ (Fig. 2 E, 2F). Seeds of *HBI1-OE* did not show the negative effect of ABA on germination (Supplementary Fig. S5), suggesting that, similar to BZR1 and BES1, HBI1, and possibly BEE2, breaks ABA-induced dormancy and promotes GA–BR-indued seed germination. Our results indicate that HBI1 and BEE2 are involved in BR-mediated seed germination by promoting endosperm rupture through controlling cell elongation.

It has been demonstrated that the tripartite HLH/bHLH module, PRE–IBH1–HBI1, regulates cell elongation in response to GA and BRs (Bai *et al.*, 2012a; Fan *et al.*, 2014). Consistent with the role of HBI1 in promoting cell elongation (Bai *et al.*, 2012a), BEE2, the closest homolog of HBI1, plays redundant roles in cell elongation (Carretero-Paulet *et al.*, 2010). The germination phenotypes of the *BEE2-OE* and HBI1*-OE* lines (Fig. 2F, G) support the individual role of HBI1 and BEE2. To identify downstream targets of HBI1 and BEE2, we performed co-expression network analysis and found that *GASA6*, known to integrate GA, ABA, and Glc signaling to regulate seed germination (Zhong *et al.*, 2015), is co-expressed with *HBI1* (Supplementary Fig. S8) and *BEE2* (Supplementary Fig. S9). In addition, similar to *HBI1* and *BEE2*, *GASA6* expression is also activated by BR and GA (Fig. 2C, D; Supplementary Fig. S2). Both HBI1 and BEE2 function in the GA- and BR-mediated seed germination processes (Fig. 2E–G). Our findings suggest that the inhibitory effect on seed germination by BRZ is overcome by *GASA6* overexpression but enhanced by *GASA6*-RNAi (Fig. 2H). We showed that HBI1 and BEE2 promote seed germination by directly regulating the expression of *GASA6*. The transcript levels of *GASA6* were significantly altered in the overexpression and repression lines of *HBI1* and *BEE2* (Fig. 3A; Supplementary Fig. S10). In addition, the region between 2.1 kb and 0.9 kb of the *GASA6* promoter is important for *GASA6* expression during seed germination and seedling growth (Fig. 3F). ChIP-qPCR, EMSA, and protoplast transactivation assays suggested that HBI1 and BEE2 activate the *GASA6* promoter mainly through binding the region between 1.4 kb and 1.1 kb (Fig. 3). Furthermore, mutations in the potential bHLH-binding motifs in the *GASA6* promoter significantly affected the promoter activity in Arabidopsis protoplasts (Fig. 3D, E), indicating that the two E-box motifs (CACATG and CATGTG) in the *GASA6* promoter are crucial for the activation by HBI1 and BEE2. *HBI1* and *BEE2* overexpression in the *gasa6* mutant increased the sensitivity to BRZ (Fig. 5). Collectively, these results indicated that *GASA6* is one of the downstream targets of HBI1 and BEE2 in the regulation of BR–GA-regulated seed germination.

Combinatorial transcriptional regulation is a hallmark of eukaryotic gene expression. Tight regulatory control is achieved by the highly dynamic nature of transcriptional activators and repressors. Heterodimeric TFs increase options of gene expression control. bHLH TFs are known to form homo- and heterodimers to regulate the expression of target genes (Toledo-Ortiz *et al.*, 2003). Here, we demonstrated the interaction between HBI1, BEE2, and IBH1 in both yeast cells and Arabidopsis mesophyll protoplasts (Fig. 4A, B). In addition, BEE2 is found to interact with HBI1 to synergistically activate *GASA6* (Fig. 4D). On the other hand, IBH1 antagonizes the function of HBI1 and BEE2 in activating *GASA6* expression, possibly by forming non-DNA-binding complexes, HBI1–IBH1 or BEE2–IBH1 (Fig. 4D). Accumulating evidence suggests that interactions between activators and repressors fine-tune plant growth, development, and metabolic outcomes. In barley, the GA pathway is controlled by the interaction of two transcriptional activators and two repressors (Zou *et al.*, 2008). In *Catharanthus roseus*, GBF1 and GBF2 interact with and antagonize transcriptional activities of MYC2 on the pathway gene promoters (Sui *et al.*, 2018). Similarly, the subgroup IIId bHLH TF RMT1 competes with MYC2 and antagonizes its activity (Patra *et al.*, 2018). In Arabidopsis, TCP4 interacts with AP2/ERF WRINKLED1 to attenuate its transcriptional activity to fine-tune seed oil accumulation (Kong *et al.*, 2020). We showed that the HBI1–BEE2–IBH1 module is critical in regulation of BR–GA-induced seed germination.

BR and GA pathways are well characterized for triggering expression of downstream genes, such as *GASA6*. Less known is how the combined effects of BRs and GA regulate the gene expression. Prior to this study, the transcriptional hub that amplifies the BR–GA signal to *GASA6* was elusive. Our findings reveal a new role for *GASA6* in BR signaling and uncover an additional molecular mechanism of GA–BR-induced seed germination in Arabidopsis. HBI1 and BEE2 promote endosperm rupture and seed germination by directly activating the expression of *GASA6*. Moreover, further dissections of the protein–protein and protein–DNA interactions associated with the regulatory network advance our understanding of GA–BR-induced cell elongation during endosperm rupture.

## Supplementary data

The following supplementary data are available at *JXB* online. 

Fig. S1. Relative gene expression of *HBI1* and *BEE2* during seed germination.

Fig. S2. Relative gene expression of *HBI1*, *BEE2*, and *GASA6* in response to BR and GA.

Fig. S3. Relative expression of *GASA6*, *HBI1*, and *BEE2* in *abi5* mutant seeds.

Fig. S4. Relative gene expression of *HBI1*, *BEE2*, and *GASA6* in response to ABA.

Fig. S5. Effect of ABA on endosperm rupture of WT, *HBI1-OE*, or *BEE2-OE* seeds.

Fig. S6. *Cis*-motif analysis of the *AtGASA6* promoter.

Fig. S7. The co-expression network of *GASA6* in Arabidopsis as analyzed by the ATTED-II network drawer.

Fig. S8. The co-expression network of *HBI1* in Arabidopsis as analyzed by the ATTED-II network drawer.

Fig. S9. The co-expression network of *BEE2* in Arabidopsis as analyzed by the ATTED-II network drawer.

Fig. S10. Relative gene expression of *GASA6* in *HBI1-* or *BEE2-OE* and *SRDX* lines.

Table S1. List of gene-specific primer sequences.

erab192_suppl_Supplementary_Materials

## Data Availability

Sequence data from this study can be found in the Arabidopsis Genome Initiative or GenBank/EMBL databases under the following accession numbers: HBI1 (At2g18300), BEE2 (At4g36540), IBH1 (At2g43060), GASA6 (At1g74670), UBQ1 (At3g52590), PP2A (At1g69960), and TUB3 (At5g 62700). All data supporting the findings of this study are available within the paper and within its supplementary data published online.
