# Supplementary material for: A transcriptional hub integrating gibberellin–brassinosteroid signals to promote seed germination in Arabidopsis
Source: J Exp Bot. 2021 May 8;72(13):4708–20. doi: 10.1093/jxb/erab192 (PMC8219041; doi:10.1093/jxb/erab192)
Supplement: erab192_suppl_Supplementary_Materials [file erab192_suppl_supplementary_materials.pdf]

Supplementary material:

A transcriptional hub integrating gibberellin-brassinosteroid signals to promote seed germination in *Arabidopsis*

Chunmei Zhong[a], Barunava Patra[c], Yi Tang[b], Xukun Li[b], Ling Yuan[c,*], and Xiaojing Wang[b,*]

[a]College of Forestry and Landscape Architecture, South China Agricultural University, Guangzhou, 510642, China

[b] Guangdong Provincial Key Laboratory of Biotechnology for Plant Development, School of Life Science, South China Normal University, Guangzhou 510631, China

[c] Department of Plant and Soil Sciences, Kentucky Tobacco Research and Development Center, University of Kentucky; Lexington, KY 40546 USA

Authors for Correspondence: Xiaojing Wang and Ling Yuan

Tel: +86 020 85216417; 001 859 257 4806

Email: wangxj@scnu.edu.cn; lyua3@uky.edu

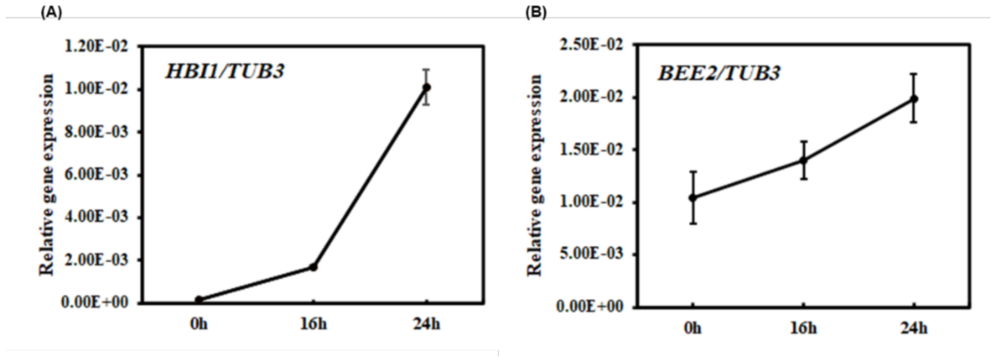

Fig. S1 Relative gene expression of *HBI1* (A) and *BEE2* (B) during seed germination, 0 h was marked as the timepoint when seeds were exposed to light after 3 days stratification. The transcript levels were normalized to *TUB3*.

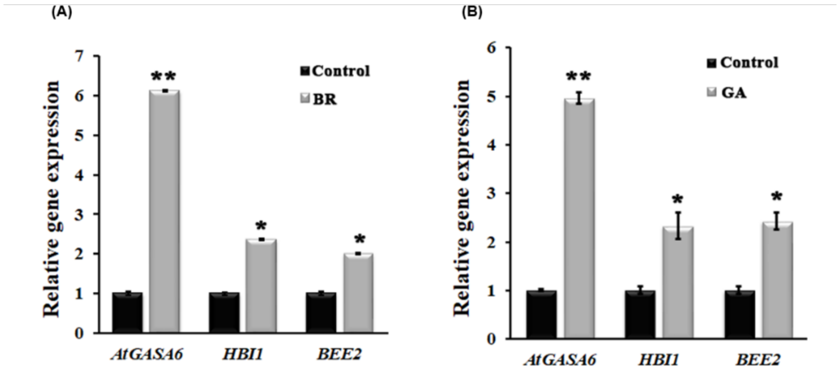

Fig. S2 Relative gene expression of *HBI1*, *BEE2*, and *GASA6* in response to BR (A) and GA (B). Two-week-old seedlings were treated with 1 uM BR or 100 uM GA_3 for 2 h. The transcript levels were normalized to *TUB3*. One-way ANOVA was used to analyze the significant difference. * $P < 0.05$; ** $P < 0.01$.

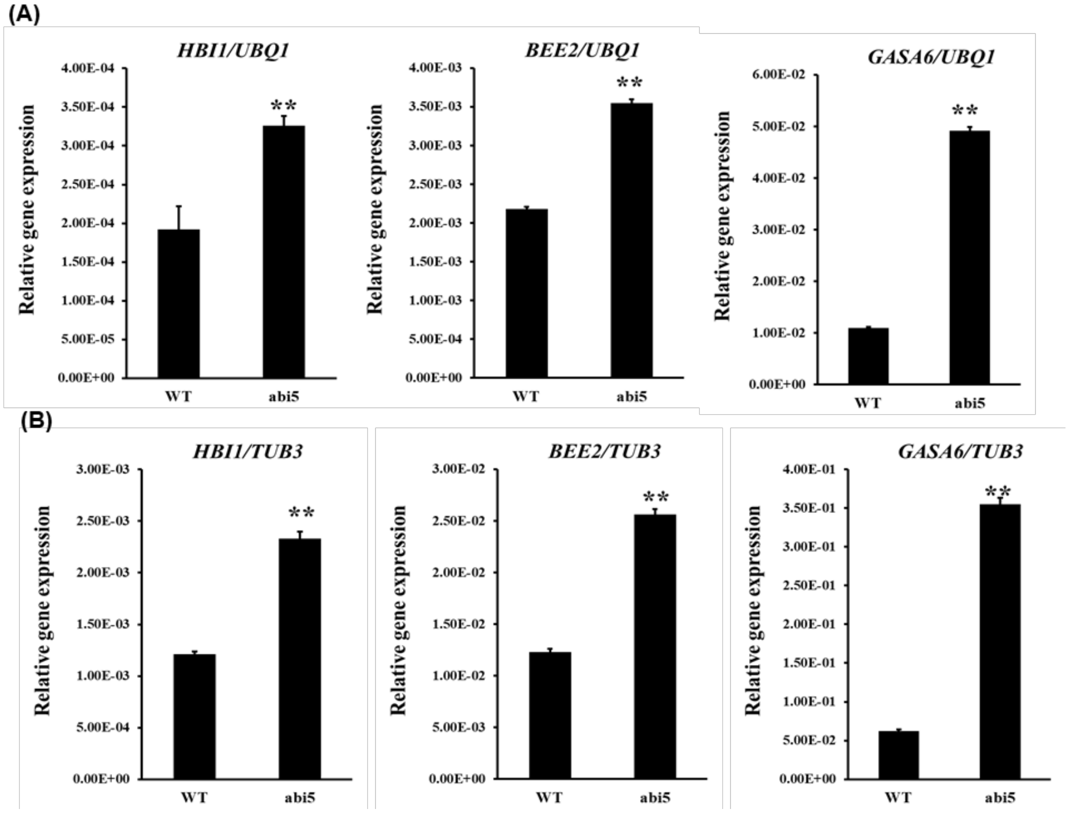

Fig. S3 Relative expression of *GASA6*, *HBI1* and *BEE2* in *abi5* mutant seeds.

Wild-type Arabidopsis (Ws) was used as the control. The transcript levels were normalized to *UBQ1* (A) or *TUB3* (B) gene expression. One-way ANOVA was used to analyze the significant difference. ** $P < 0.01$.

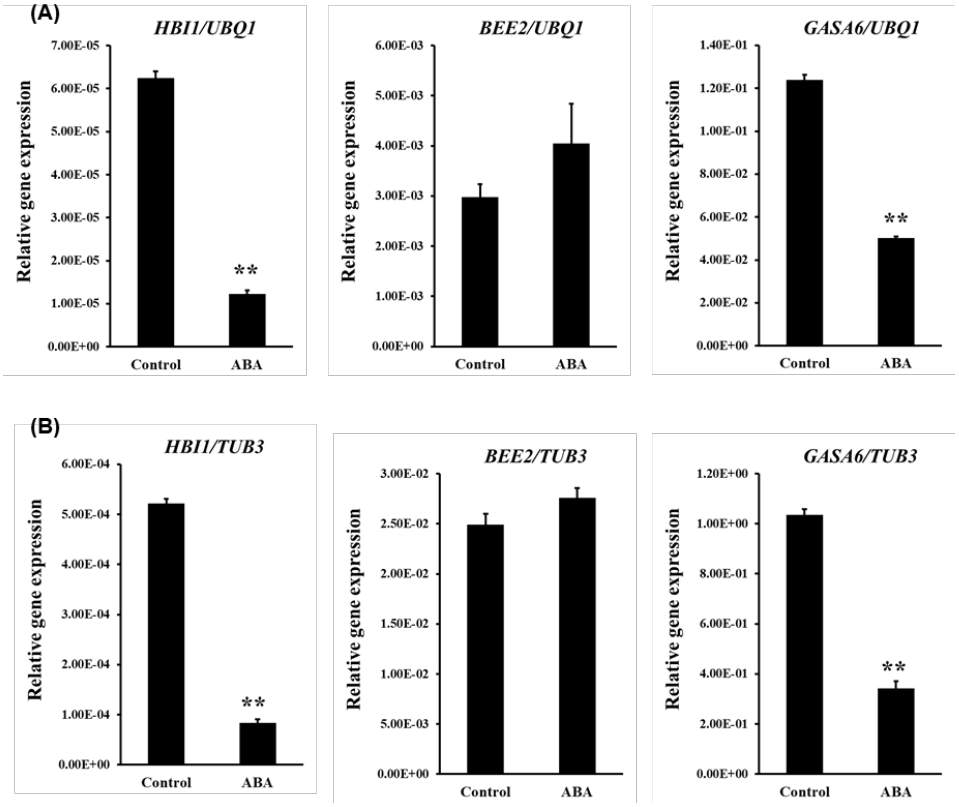

Fig. S4 Relative gene expression of *HBI1*, *BEE2*, and *GASA6* in response to ABA. Germinating seeds were treated with 100 uM ABA for 2 h. The transcript levels were normalized to *UBQ1* (A) or *TUB3* (B) gene expression. One-way ANOVA was used to analyze the significant difference. ** $P < 0.01$.

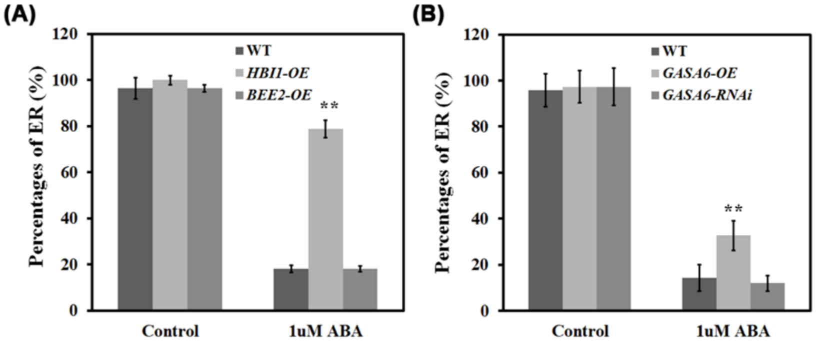

Fig. S5 Effect of ABA on ER of WT, *HBI1-OE* or *BEE2-OE* seeds.

(A) Percentage of ER in WT, *HBI1-OE* or *BEE2-OE*, seeds were treated with 1 μM ABA for 36 h. (B) Percentage of ER in WT, GASA6-OE and RNAi seeds treated with 1 μM ABA for 36 h. Seeds germinated on ½ basal MS was used as all of the control. The asterisks indicate significant differences compared with WT. one-way ANOVA was used to analyze the significant difference. ** $P < 0.01$.

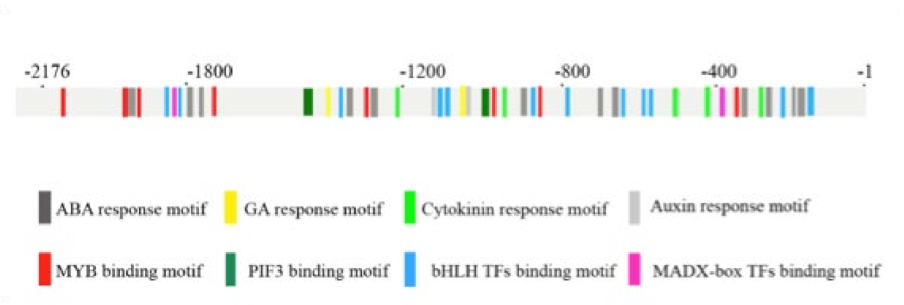

Fig. S6 *Cis*-motif analysis of *AtGASA6* promoter. Distribution of ABA-, GA-, CK- and Auxin-response elements, and MYB, PIF, bHLH and MADX-box transcription factors binding elements in *AtGASA6* promoter. Cis-motifs were identified by using online software PlantPAN (http://plantpan2.itps.ncku.edu.tw/), PLACE (http://www.dna.affrc.go.jp/PLACE/) and AGRIS (http://arabidopsis.med.ohio-state.edu/). Promoter of *AtGASA6* 2176 bp from ATG was used for analysis.

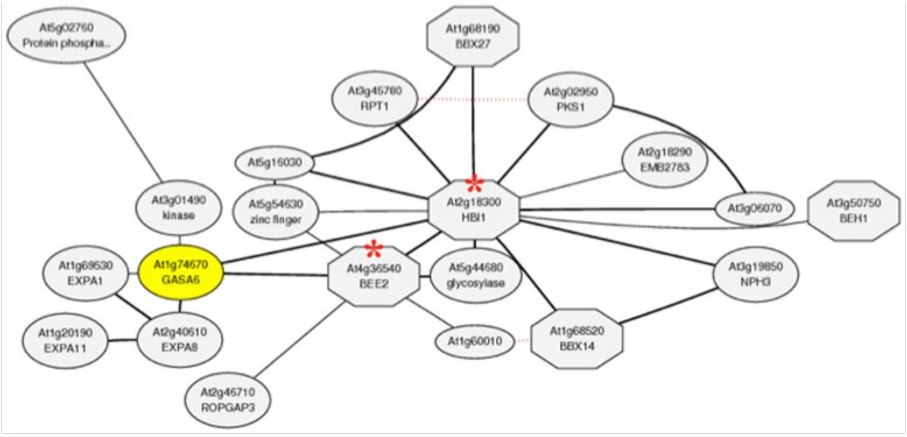

Fig. S7. The co-expression network of *AtGASA6* in Arabidopsis analyzed by the ATTED-II network drawer (http://atted.jp/top_draw.shtml#NetworkDrawer). The yellow oval indicated the query gene. The red asterisks indicate the closet genes.

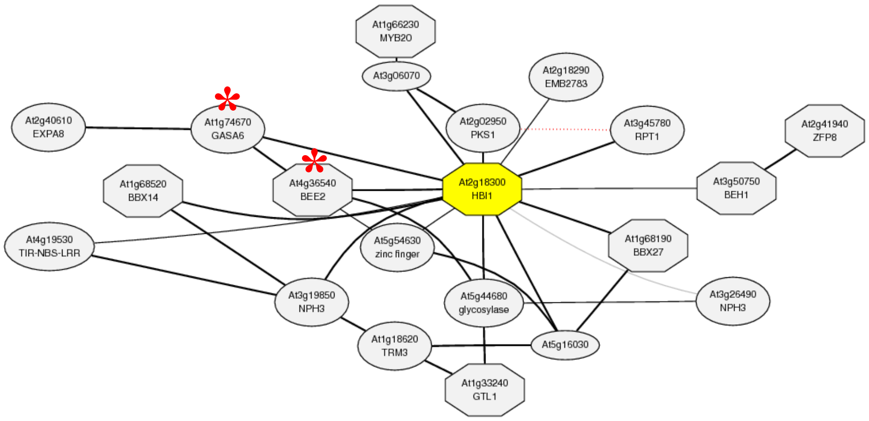

Fig. S8. The co-expression network of *HBI1* in Arabidopsis analyzed by the ATTED-II network drawer. The yellow oval indicated the query gene. The red asterisks indicate the closet genes.

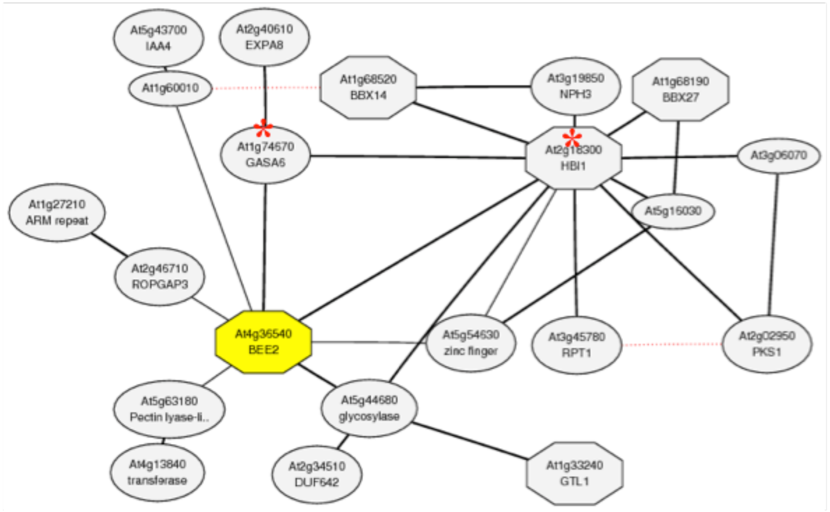

Fig. S9. The co-expression network of *BEE2* in Arabidopsis analyzed by the ATTED-II network drawer. The yellow oval indicated the query gene. The red asterisks indicate the closet genes.

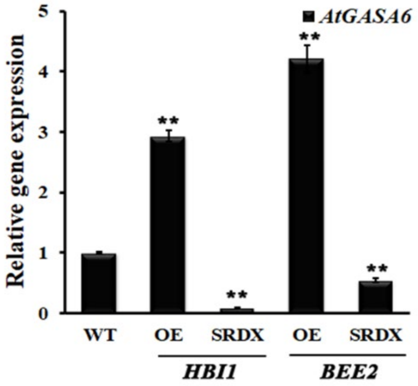

Fig. S10 Relative gene expression of *GASA6* in *HBI1*- or *BEE2*-OE and SRDX lines. Col0 was used as WT. The transcript levels were normalized to *TUB3*. One-way ANOVA was used to analyze the significant difference. ** $P < 0.01$.

Table S1 List of gen-specific primer sequences

Primer name	Sequence (5' to 3')
GASA6-FW-QPCR	CAATGCACAAGGAGATGTAGCAAC
GASA6-RV-QPCR	AGGGACACAAAGGCATTTAGCA
HBI1-FW-QPCR	TGTGCCTGGATGCAATAAGG
HBI1-RV-QPCR	ATACATCTTCCACGGCAAGC
BEE2-FW-QPCR	ACAGAGCCAAGCATGAAAGG
BEE2-RV-QPCR	TTGCCCTCTCTGCTAAGCTATG
UBQ1-FW-QPCR	GGCCAAGATCCAAGACAAAG
UBQ1-RV-QPCR	GTTGACAGCTCTTGGGTGAA
TUB3-FW-QPCR	GTGACTGCCTCCAAGGGTTC
TUB3-RV-QPCR	CGTTGTAAGGCTCCACCACA
PP2A-ChIP-F	ATGGCTGCGATTCTAGAGATCG
PP2A-ChIP-R	TAATCTGGAGTCTTGCGAGTGG
GASA6-P1-ChIP-F-2	GAATGACTTAAACAATCAAGTGGCAG
GASA6-P1-ChIP-R-2	GTGTGTGTAAGCTTCCTCATTCAC
GASA6-P2- ChIP-F-2	GACACTATTAGTATATTATTGGACCCG
GASA6-P2- ChIP-R-2	CTACAGGCAGAAATTATTTTACATGTG

GASA6-P3- ChIP-F-1	TGGTCCCAATCACTTGCACT
GASA6-P3- ChIP-R-1	GTCACATTTGGGACTACACCC
GASA6-P4- ChIP-F-1	GTGTATCCACAAAATGCAAAGATAGA
GASA6-P4- ChIP-R-1	GAGAGACAAAAGGGTAGAGGCA
GASA6-P5- ChIP-F-1	GAATTGTGAGTGTCGTAACAAGAT
GASA6-P5- ChIP-R-1	TTGGTCGTCCAATAAAATAGCATT
GASA6-P6- ChIP-F-1	CATCTCCATCAATGGTTTCTCATTG
GASA6-P6- ChIP-R-1	ACAAGTGTTCTTATGGGTTTCTCAG
pGASA6-1.4k-LUC-XhoI-F	CCGCTCGAGCTTTGAATTGTGAGTGTCGT
pGASA6-1.4k-LUC-PstI-R	CAACTGCAGGACTAGCTCTTGAGAGAGAGAG
pGASA6-1.2k-LUC- XhoI -F	CCGCTCGAGCCACAAAATGCAAAGATAGAA
pGASA6-1.1k-LUC- XhoI -F	CCGCTCGAGAAACTGCCTCTACCCTTTTGTCT
pGASA6-0.9k-LUC- XhoI -F	CCGCTCGAGTATTTGTTGGTTGGTCCCAATC
pGASA6-1.4k-mut1-LUC-F	CACAAAAACTATTTTATTGGACG
pGASA6-1.4k-mut1-LUC-R	ATTAAAAATTGTTTGTGTTTAACG
pGASA6-1.4k-mut2-LUC-F	CCATAAAAGT GCACATGGAGTT
pGASA6-1.4k-mut2-LUC-R	GAGACATATATCTATCTAATGCTTTC
pGASA6-1.4k-mut3-LUC-F	CCATGTGAGTGCACAAAGAGTT
pGASA6-1.4k-mut3-LUC-R	The same sequence as *pGASA6*-1.4k-mut2-LUC-R
HBI1-FW-PBS-BamHI	GTACGGATCCATGTTGGAAGGTCTTGTCTCTCA
HBI1-RV-PBS-PstI	ATGCTGCAGTTAGTAATGAAAACCGAGGCTAG
BEE2-FW-PBS-BamHI	AGCTGGATCCATGGACTTGTCTGTACTTGATAGGC
BEE2-RV-PBS-XbaI	GTACTCTAGATTACTTGAGGCTGAAGAAATTGGA
IBH1-FW-PBS-BamHI	ATCGGGATCCATGGCCTCTGCAGACAAACT
IBH1-RV-PBS-XbaI	ATCGTCTAGAATCATTTGGGAGATAAGCCATCA
HBI1-pAD/BD-EcoRI-F	ACCGGAATTCATGTTGGAAGGTCTTGTCTCTCA
HBI1-pAD-XhoI-R	CACGCTCGAGTTAGTAATGAAAACCGAGGCTAG
HBI1-pBD-PstI-R	CCAACTGCAGTTAGTAATGAAAACCGAGGCTAG

BEE2-pAD-BamHI-F	ACGCGGATCCATATGGACTTGTCTGTACTTGATAGGC
BEE2-pAD-XhoI-R	CACGCTCGAGTTACTTGAGGCTGAAGAAATTG
BEE2-pBD-NcoI-F	CACGCCATGGAGATGGACTTGTCTGTACTTGATAGGC
BEE2-pBD-BamHI-R	ACGCGGATCCTTACTTGAGGCTGAAGAAATTG
IBH1-pAD/pBD-EcoRI-F	ACCGGAATTCATGGCCTCTGCAGACAAACT
IBH1-pAD/pBD-BamHI-R	ACGCGGATCCTCATTTGGGAGATAAGCCATCA
HBI1-BiFC-EcoRI-F	ACCGGAATTCTATGTTGGAAGGTCTTGTCTCTCA
HBI1-BiFC-BamHI-R	CGCGGATCCTTAGTAATGAAAACCGAGGCTAG
BEE2-BiFC-XhoI-F	CCGCTCGAGCTATGGACTTGTCTGTACTTGATAGGC
BEE2-BiFC-BamHI-R	The same sequence as BEE2-pBD-BamHI-R
IBH1-BiFC-EcoRI-F	ACCGGAATTCTATGGCCTCTGCAGACAAACT
IBH1-BiFC-BamHI-R	The same sequence as IBH1-pAD/pBD-BamHI-R
BEE2-pAD-N1-BamHI-F	ACGGGATCCATATGGACTTGTCTGTACTTGATAGGC
BEE2-pAD-N1-XhoI-R	TCCGCTCGAGTTCTGGATTTATGACAGAGAGTTTCA
BEE2-pAD-N2-BamHI-F	The same sequence as BEE2-pAD-N1-BamHI-F
BEE2-pAD-N2-XhoI-R	TCCGCTCGAGGGCTTCACCTCGTCTAGCC
BEE2-pAD-C1-BamHI-F	ACGCGGATCCATACCGACAGACATAGCTTAGCAG
BEE2-pAD-C1-XhoI-R	TCCGCTCGAGTTACTTGAGGCTGAAGAAATTGGA
BEE2-pAD-C2-BamHI-F	CGCGGATCCATCTTGAGTGTCATATCGATGATTT
BEE2-pAD-C2-XhoI-R	The same sequence as BEE2-pAD-C1-XhoI-R
ProGASA6-GUS-a-F	GCGTCGACGGTAAAGGGTTTATCAAGTAAG
ProGASA6-GUS-b-F	GCGTCGACAGTCGTTATGTTTGTTGAAAGC
ProGASA6-GUS-c-F	ATGTCGACTATTTGTTGGTTGGTCCCAATCAC
ProGASA6-GUS-d-F	TCGTCGACTGCCTGTAGTCTTTTAATCACTG
ProGASA6-GUS-R	TACCCGGGCATGACTAGCTCTTGAGAGAGAG
Probe -1	CAATTTTTAATCACAAATGCTATTTTATTGGACGACC
Probe 2	GTCTCC*CATGTG*AGTG*CACATG*GAGTTATG
